# Supplementary material for: Modulating Gastrointestinal Microbiota in Preweaning Dairy Calves: Dose-Dependent Effects of Milk-Based Sodium Butyrate Supplementation
Source: Microorganisms. 2024 Feb 5;12(2):333. doi: 10.3390/microorganisms12020333 (PMC10893347; doi:10.3390/microorganisms12020333)
Supplement: Supplementary file 1 [file microorganisms-12-00333-s001.zip › microorganisms-2811650-supplementary.pdf]

Article

Modulating Gastrointestinal Microbiota in Preweaning Dairy Calves: Dose-Dependent Effects of Milk-Based Sodium Butyrate Supplementation

Donglin Wu [1], Zhanhe Zhang [1], Qifan Song [1], Yang Jia [1,2], Jingwei Qi [1,2], and Ming Xu [1,2,*]

[1] College of Animal Science, Inner Mongolia Agricultural University, Hohhot 010018, China; wdl2017@emails.imau.edu.cn (D.W.); nddky@emails.imau.edu.cn (Z.Z.); jiayang@imau.edu.cn (Y.J.)

[2] National Center of Technology Innovation for Dairy, Hohhot, Inner Mongolia 010080, China

* Correspondence: ndxm@imau.edu.cn;
Tel.: +86-1362-4710-492; Fax: +86-471-4309-170

Supplementary material – Tables and Figures

Supplemental Table S1. Information on the sequencing depth of 16S rRNA genes in ruminal microbiota.

Sample ID	Total read bases (bp)	Clean reads	Mean_length	Min_length	Max_length	Goods -coverage
CON1	16256853	39095	415.829467	233	478	0.998637
CON2	16044873	38735	414.221583	216	462	0.999215
CON3	21570013	51994	414.85581	217	465	0.999009
CON4	20431997	48788	418.791445	210	469	0.998679
CON5	25350461	60844	416.646851	234	432	0.998926
CON6	18224940	43841	415.70539	216	518	0.998803
CON7	17619410	42338	416.160659	204	434	0.998637
CON8	19853293	48220	411.723206	219	465	0.99872
CON9	24377464	58619	415.862843	214	480	0.998968
CON10	20263955	48736	415.790278	210	469	0.998968
LSB1	17991816	43356	414.978688	265	496	0.998596
LSB2	19864574	47626	417.095158	216	531	0.998514
LSB3	19120315	45832	417.182645	252	528	0.999009
LSB4	22767759	54946	414.366087	219	489	0.998803
LSB5	26920641	64834	415.224126	235	436	0.999009
LSB6	24281196	58193	417.252865	250	437	0.998596
LSB7	17411582	41998	414.581218	248	481	0.999174
LSB8	22639823	54046	418.899141	235	438	0.998514
LSB9	24560953	58726	418.229626	201	434	0.998803
LSB10	18878391	45372	416.080204	230	469	0.998844
MSB1	14158134	34022	416.146435	278	473	0.998761
MSB2	23554526	57096	412.54249	219	474	0.998472
MSB3	21820955	52726	413.855688	251	476	0.998761
MSB4	16272903	39244	414.659642	219	455	0.998348
MSB5	26108834	62958	414.702405	205	498	0.998926
MSB6	15460972	37456	412.776912	227	471	0.999257
MSB7	19741962	47509	415.541518	233	505	0.999009
MSB8	29258255	70139	417.146737	210	471	0.998926
MSB9	29453352	71027	414.678249	219	491	0.999298
MSB10	18237598	44001	414.481444	232	511	0.998844
HSB1	17692281	42679	414.543007	219	431	0.999174
HSB2	25383842	61090	415.515502	201	484	0.998844
HSB3	26529525	63760	416.084144	222	472	0.999092
HSB4	17628910	42704	412.816364	263	452	0.998885
HSB5	21695051	52180	415.773304	232	431	0.999009
HSB6	21402007	51251	417.591988	205	513	0.998968
HSB7	27136051	64891	418.178962	213	431	0.999009
HSB8	31163496	74227	419.840435	232	493	0.999215
HSB9	23928558	57637	415.159672	216	518	0.998968
HSB10	23218823	55744	416.525958	213	463	0.998968
Summation		2078480				
Average	21606908.6	51962	415.7009537	225.825	473.1	0.99887895

CON represents the control group, which received milk without sodium butyrate (SB) supplementation. LSB refers to the low SB supplementation group, MSB refers to the medium SB supplementation group, and HSB refers to the high SB supplementation group.

Supplemental Table S2. Effects of sodium butyrate supplementation on taxonomic analysis (> 0.1%) of ruminal microbiota at the phylum and genus levels in preweaning calves.

Items	Supplementation level, g/d				SEM	*p*-Value		
	0	4.4	8.8	17.6		ANOVA	Linear	Quadratic
Phylum level								
Firmicutes	46.92	45.49	50.67	46.97	1.83	0.777	0.845	0.600
Bacteroidota	27.42	32.26	26.36	36.72	1.93	0.209	0.137	0.452
Actinobacteriota	21.89	15.18	19.30	11.19	1.69	0.117	0.047	0.884
Spirochaetota	1.20	2.29	0.44	1.65	0.345	0.291	0.998	0.642
Synergistota	0.891	1.50	0.848	1.04	0.211	0.694	0.913	0.799
Patescibacteria	0.537	0.813	1.31	1.17	0.254	0.718	0.365	0.529
Proteobacteria	0.472	0.544	0.500	0.619	0.122	0.979	0.709	0.945
Desulfobacterota	0.440	0.493	0.399	0.512	0.049	0.858	0.714	0.710
Genus level								
Firmicutes phylum								
Succiniclasticum	5.08	6.19	7.58	4.86	0.643	0.434	0.857	0.119
Shuttleworthia	3.27	2.81	3.93	4.41	0.682	0.858	0.464	0.896
Lachnospiraceae_NK3A20_group	3.81	3.27	3.27	3.79	0.328	0.895	0.914	0.456
Acetitomaculum	3.86	2.30	3.97	3.22	0.475	0.596	0.937	0.858
Sharpea	3.22	2.01	0.98	2.92	0.387	0.164	0.883	0.029
Syntrophococcus	2.16	1.77	2.06	1.46	0.155	0.393	0.163	0.776
Erysipelotrichaceae_UCG-002	2.29	1.66	0.89	0.42	0.459	0.501	0.143	0.704
Ruminococcus_gauvreauii_group	1.30	2.60	1.21	0.69	0.263	0.061	0.117	0.230
NK4A214_group	1.50	1.15	1.27	1.48	0.162	0.861	0.869	0.460
Eubacterium_nodatum_group	1.38	1.32	1.49	1.07	0.125	0.705	0.424	0.494
Ruminococcus	0.759	0.864	0.864	1.16	0.188	0.900	0.469	0.880
Christensenellaceae_R-7_group	0.605	0.494	1.42	0.533	0.263	0.572	0.938	0.340
Oscillospiraceae_UCG-002	0.259[b]	0.552[b]	0.432[b]	1.80[a]	0.210	0.031	0.007	0.291
Oribacterium	0.502	0.837	0.642	0.600	0.128	0.835	0.995	0.551

Coprococcus	0.399	0.386	0.519	0.476	0.061	0.854	0.564	0.739
Acidaminococcus	0.655	0.340	0.624	0.134	0.099	0.197	0.107	0.650
Roseburia	0.335	0.429	0.324	0.611	0.085	0.627	0.293	0.614
Lachnospiraceae_FE2018_group	0.509	0.316	0.218	0.332	0.045	0.144	0.214	0.049
Pseudoramibacter	0.347	0.255	0.230	0.161	0.026	0.075	0.012	0.538
Lachnoclostridium	0.233	0.206	0.202	0.246	0.026	0.929	0.797	0.539
Moryella	0.232	0.178	0.193	0.191	0.020	0.803	0.604	0.528
Catenisphaera	0.272	0.199	0.109	0.186	0.028	0.239	0.275	0.098
Erysipelotrichaceae_UCG-006	0.225	0.117	0.113	0.144	0.020	0.183	0.283	0.069
Mogibacterium	0.113	0.143	0.137	0.134	0.012	0.842	0.679	0.503
Solobacterium	0.116	0.108	0.181	0.105	0.027	0.729	0.987	0.429
Bacteroidota phylum								
Prevotella	20.18	22.00	17.76	29.70	1.88	0.127	0.078	0.192
Rikenellaceae_RC9_gut_group	2.08	3.39	2.72	0.999	0.438	0.262	0.211	0.149
Prevotellaceae_UCG-001	0.318	0.539	0.697	0.630	0.119	0.709	0.385	0.438
Prevotellaceae_YAB2003_group	0.180	0.278	0.137	0.142	0.047	0.713	0.558	0.841
Actinobacteriota phylum								
Olsenella	19.83	13.31	17.54	10.14	1.62	0.149	0.068	0.918
Atopobium	0.580	0.701	0.913	0.152	0.225	0.691	0.479	0.359
Bifidobacterium	0.754	0.446	0.458	0.407	0.108	0.666	0.344	0.500
Spirochaetota phylum								
Treponema	0.871	1.95	0.216	1.19	0.337	0.341	0.895	0.764
Sphaerochaeta	0.334	0.344	0.228	0.464	0.050	0.432	0.389	0.246
Synergistota phylum								
Pyramidobacter	0.341	0.495	0.346	0.295	0.086	0.869	0.682	0.691
Desulfobacterota phylum								
Desulfovibrio	0.437	0.484	0.391	0.501	0.049	0.870	0.747	0.696

SEM = standard error of the means; $n = 10$ for each group; Means are significantly different with $p < 0.05$.

Supplemental Table S3. Information on the sequencing depth of 16S rRNA genes in intestinal microbiota.

Sample ID	Total read bases (bp)	Clean reads	Mean_length	Min_length	Max_length	Goods -coverage
CON1	21266760	51032	416.733814	337	431	0.997651
CON2	23884539	57457	415.694154	307	431	0.996434
CON3	20907343	50672	412.601496	245	503	0.995218
CON4	20185857	48651	414.911451	270	431	0.996728
CON5	14884604	35841	415.295444	277	472	0.995973
CON6	20111360	48904	411.241616	284	432	0.996141
CON7	22469061	53834	417.376769	246	438	0.995511
CON8	20497535	49731	412.168165	270	430	0.99677
CON9	20664942	49537	417.161758	216	432	0.995889
CON10	20822587	49764	418.426714	270	468	0.995008
LSB1	17911722	41921	427.273252	270	431	0.998448
LSB2	25752344	62127	414.511307	216	431	0.996225
LSB3	22602244	54143	417.454592	216	431	0.995595
LSB4	19660791	47482	414.0683	218	491	0.995973
LSB5	21188240	51152	414.221145	271	446	0.99677
LSB6	21047564	50782	414.468985	215	493	0.996308
LSB7	20349611	48701	417.847909	232	471	0.996728
LSB8	22249705	53423	416.481759	287	494	0.994882
LSB9	18910275	46156	409.703506	267	472	0.99635
LSB10	21625147	51833	417.208091	270	511	0.995302
MSB1	20026723	48438	413.450659	216	432	0.995973
MSB2	23763479	57398	414.012318	216	458	0.995931
MSB3	18648569	45160	412.944398	216	432	0.996434
MSB4	20285953	48945	414.464256	255	431	0.995469
MSB5	20561792	49670	413.968029	249	509	0.995763
MSB6	21914346	52651	416.218989	214	431	0.996476
MSB7	19884165	47752	416.404863	292	493	0.995427
MSB8	19554053	47162	414.614584	221	432	0.997399
MSB9	21647865	51865	417.388701	252	497	0.995553
MSB10	17645922	42342	416.747485	215	467	0.996518
HSB1	23922184	57520	415.893324	235	465	0.995763
HSB2	19765261	48012	411.673352	252	432	0.997231
HSB3	17982247	43246	415.812954	219	441	0.997231
HSB4	17960105	43040	417.288685	271	431	0.995427
HSB5	21841288	52801	413.652923	248	443	0.995889
HSB6	19829928	47515	417.340377	271	499	0.997357
HSB7	20437373	48747	419.253964	210	438	0.996812
HSB8	20120400	48488	414.956278	230	509	0.996896
HSB9	20721154	50277	412.139825	317	489	0.995931
HSB10	19544254	46836	417.291272	252	485	0.996686
Summation		1981008				
Average	20576232.3	49525.2	415.5091866	250.875	458.825	0.99625175

CON represents the control group, which received milk without sodium butyrate (SB) supplementation. LSB refers to the low SB supplementation group, MSB refers to the

medium SB supplementation group, and HSB refers to the high SB supplementation group.

Supplemental Table S4. Effects of sodium butyrate supplementation on taxonomic analysis (> 0.1%) of intestinal microbiota at the phylum and genus levels in preweaning calves.

Items	Supplementation level, g/d				SEM	*p*-Value		
	0	4.4	8.8	17.6		ANOVA	Linear	Quadratic
Phylum level								
Firmicutes	61.02	63.21	54.19	53.11	1.89	0.156	0.059	0.890
Bacteroidota	35.32	29.42	38.28	39.99	2.03	0.277	0.195	0.643
Actinobacteriota	1.70	3.15	3.90	2.85	0.648	0.701	0.604	0.290
Proteobacteria	0.655	3.200	0.810	0.834	0.413	0.083	0.531	0.287
Patescibacteria	0.141	0.133	1.39	2.61	0.505	0.253	0.054	0.848
Spirochaetota	0.523	0.413	0.649	0.267	0.127	0.760	0.551	0.570
Cyanobacteria	0.106	0.118	0.523	0.228	0.097	0.400	0.522	0.254
Genus level								
Bacteroidota phylum								
Bacteroides	13.00	7.63	5.66	12.62	1.41	0.169	0.871	0.027
Rikenellaceae_RC9_gut_group	4.16[ab]	3.26[b]	7.41[a]	7.29[a]	0.670	0.048	0.027	0.666
Alloprevotella	1.90	3.12	2.98	4.06	0.580	0.641	0.234	0.850
Parabacteroides	3.02	2.15	3.48	3.23	0.385	0.656	0.599	0.934
Odoribacter	1.24	0.91	1.27	2.41	0.238	0.121	0.039	0.224
Prevotellaceae_NK3B31_group	1.88	1.25	0.64	1.81	0.370	0.624	1.000	0.201
Prevotella	1.12	0.716	0.961	0.617	0.191	0.792	0.455	0.936
Alistipes	0.293	0.292	0.412	0.628	0.089	0.517	0.149	0.746
Prevotellaceae_UCG-003	0.417	0.288	0.242	0.305	0.067	0.834	0.629	0.435
Butyricimonas	0.158	0.194	0.221	0.430	0.059	0.378	0.095	0.635
Firmicutes phylum								
Lactobacillus	9.96	11.44	6.96	5.41	1.26	0.317	0.109	0.881
UCG-005	6.20	9.83	7.87	7.77	1.09	0.719	0.854	0.472
Blautia	6.04	6.31	4.65	4.95	0.545	0.663	0.368	0.742
Faecalibacterium	5.22[a]	2.30[b]	3.36[b]	4.79[ab]	0.415	0.043	0.741	0.015

Christensenellaceae_R-7_group	1.85	2.11	2.34	2.13	0.365	0.975	0.804	0.708
Lachnoclostridium	1.67	1.48	1.52	1.42	0.141	0.938	0.598	0.846
Ruminococcus	1.17	0.461	1.87	0.729	0.296	0.368	0.873	0.501
Ruminococcus_torques_group	1.03	0.801	0.941	1.01	0.142	0.944	0.906	0.675
Phascolarctobacterium	0.572	1.35	0.495	1.10	0.230	0.501	0.675	0.983
Subdoligranulum	0.520	1.02	0.643	0.485	0.176	0.706	0.692	0.482
Ruminococcus_gauvreauii_group	0.559	0.567	0.555	0.435	0.096	0.961	0.634	0.804
Romboutsia	0.745	0.397	0.642	0.298	0.141	0.669	0.371	0.994
Butyricicoccus	0.449	0.256	0.101	0.316	0.063	0.277	0.519	0.070
Monoglobus	0.395	0.226	0.209	0.170	0.036	0.131	0.048	0.243
Lachnospiraceae_NK4A136_group	0.243	0.425	0.227	0.098	0.084	0.603	0.359	0.533
Negativibacillus	0.211	0.349	0.133	0.234	0.039	0.267	0.757	0.847
Marvinbryantia	0.222	0.243	0.145	0.307	0.055	0.793	0.645	0.502
Fournierella	0.250	0.315	0.121	0.137	0.030	0.059	0.052	0.773
NK4A214_group	0.184	0.205	0.294	0.139	0.051	0.767	0.777	0.369
Oscillospira	0.246	0.250	0.120	0.201	0.042	0.687	0.595	0.475
Colidextribacter	0.300	0.186	0.133	0.193	0.036	0.426	0.356	0.168
Erysipelatoclostridium	0.211	0.183	0.162	0.105	0.037	0.794	0.317	0.973
Eubacterium_nodatum_group	0.296	0.169	0.129	0.056	0.044	0.280	0.067	0.557
Actinobacteriota phylum								
Collinsella	0.885	2.154	1.947	2.172	0.541	0.823	0.506	0.608
Bifidobacterium	0.206	0.086	0.991	0.182	0.227	0.480	0.865	0.304
Olsenella	0.408	0.370	0.432	0.191	0.058	0.467	0.198	0.445
Proteobacteria phylum								
Sutterella	0.476	0.294	0.253	0.591	0.091	0.536	0.538	0.185
Escherichia-Shigella	0.150	0.812	0.505	0.063	0.153	0.289	0.504	0.123
Spirochaetota phylum								
Treponema	0.485	0.357	0.359	0.232	0.112	0.896	0.466	0.931

SEM = standard error of the means; $n = 10$ for each group; Means are significantly different with $p < 0.05$.

Supplemental Table S5. Detailed information on the relationship between ruminal microbiota and intestinal microbiota.

r	ISobs	IShannon	ISimpson	IAce	IChao1	IShannoneven	ISimpsoneven	IPd	*p*-Value	ISobs	IShannon	ISimpson	IAce	IChao1	IShannoneven	ISimpsoneven	IPd
CON																	
RSobs	-0.572	-0.407	0.307	-0.450	-0.570	-0.309	0.019	-0.595	RSobs	0.0838	0.2432	0.3877	0.1919	0.0855	0.3854	0.9586	0.0698
RShannon	0.115	0.198	-0.289	0.200	0.135	0.228	0.276	0.132	RShannon	0.7509	0.5840	0.4175	0.5792	0.7110	0.5264	0.4407	0.7157
RSimpson	-0.192	-0.191	0.253	-0.304	-0.251	-0.181	-0.159	-0.220	RSimpson	0.5955	0.5969	0.4811	0.3930	0.4834	0.6164	0.6614	0.5405
RAce	-0.350	-0.256	0.194	-0.239	-0.359	-0.191	0.162	-0.424	RAce	0.3208	0.4748	0.5916	0.5068	0.3079	0.5973	0.6547	0.2220
RChao1	-0.521	-0.388	0.286	-0.451	-0.555	-0.307	0.104	-0.540	RChao1	0.1226	0.2680	0.4233	0.1909	0.0962	0.3890	0.7757	0.1074
RShannoneven	0.308	0.347	-0.419	0.368	0.333	0.349	0.299	0.335	RShannoneven	0.3870	0.3266	0.2280	0.2955	0.3477	0.3228	0.4007	0.3441
RSimpsoneven	0.495	0.496	-0.539	0.568	0.544	0.476	0.344	0.536	RSimpsoneven	0.1462	0.1448	0.1083	0.0865	0.1040	0.1645	0.3300	0.1105
RPd	-0.645	-0.498	0.388	-0.517	-0.615	-0.404	-0.018	-0.648	RPd	0.0442	0.1431	0.2681	0.1263	0.0585	0.2475	0.9603	0.0426
LSB																	
RSobs	0.021	-0.051	0.073	0.061	0.081	-0.092	-0.191	0.050	RSobs	0.9533	0.8880	0.8402	0.8672	0.8232	0.8013	0.5976	0.8904
RShannon	0.80	0.828	-0.832	0.78	0.799	0.788	0.295	0.82	RShannon	0.004	0.0031	0.0028	0.007	0.0055	0.0068	0.4084	0.003

	6			6				8		9			0				1
RSimpson	-0.812	-0.895	0.925	-0.768	-0.778	-0.877	-0.408	-0.822	RSimpson	0.0043	0.0005	0.0001	0.0094	0.0080	0.0009	0.2419	0.0035
RAce	0.323	0.214	-0.140	0.402	0.421	0.157	0.031	0.305	RAce	0.3622	0.5533	0.6990	0.2489	0.2254	0.6639	0.9316	0.3917
RChao1	0.161	0.063	-0.018	0.227	0.249	0.013	-0.110	0.173	RChao1	0.6563	0.8633	0.9615	0.5291	0.4880	0.9709	0.7631	0.6322
RShannoneven	0.866	0.916	-0.929	0.830	0.837	0.886	0.382	0.880	RShannoneven	0.0012	0.0002	0.0001	0.0030	0.0025	0.0006	0.2765	0.0008
RSimpsoneven	0.707	0.827	-0.869	0.643	0.645	0.837	0.493	0.696	RSimpsoneven	0.0222	0.0031	0.0011	0.0448	0.0442	0.0025	0.1477	0.0253
RPd	-0.084	-0.100	0.095	-0.032	-0.011	-0.109	-0.069	-0.052	RPd	0.8166	0.7840	0.7933	0.9293	0.9760	0.7639	0.8495	0.8855
MSB																	
RSobs	-0.349	-0.224	0.085	-0.382	-0.397	-0.172	0.105	-0.387	RSobs	0.323	0.534	0.816	0.276	0.257	0.636	0.772	0.270
RShannon	0.039	0.148	-0.148	0.011	-0.021	0.159	0.525	-0.005	RShannon	0.914	0.683	0.684	0.976	0.955	0.660	0.120	0.990
RSimpson	-0.062	-0.160	0.121	-0.032	0.019	-0.166	-0.506	-0.065	RSimpson	0.864	0.659	0.739	0.929	0.959	0.646	0.135	0.858
RAce	-0.272	-0.069	-0.059	-0.253	-0.201	-0.016	0.192	-0.309	RAce	0.447	0.849	0.871	0.481	0.577	0.966	0.595	0.386
RChao1	-	-0.157	0.009	-	-0.391	-0.095	0.094	-	RChao1	0.313	0.664	0.981	0.268	0.264	0.794	0.795	0.339

	0.356			0.388				0.338									
RShannoneven	0.139	0.236	-0.200	0.115	0.080	0.236	0.597	0.097	RShannoneven	0.701	0.511	0.580	0.752	0.827	0.511	0.068	0.789
RSimpsoneven	0.260	0.368	-0.282	0.231	0.177	0.358	0.700	0.265	RSimpsoneven	0.469	0.296	0.430	0.521	0.626	0.310	0.024	0.459
RPd	-0.309	-0.108	-0.054	-0.340	-0.313	-0.048	0.140	-0.313	RPd	0.384	0.767	0.883	0.337	0.379	0.896	0.699	0.379
HSB																	
RSobs	0.245	0.104	-0.157	0.189	0.161	0.014	-0.159	0.243	RSobs	0.4958	0.7748	0.6651	0.6006	0.6573	0.9694	0.6603	0.4990
RShannon	0.079	-0.028	-0.153	0.016	-0.019	-0.067	-0.049	0.074	RShannon	0.8289	0.9397	0.6727	0.9645	0.9580	0.8537	0.8926	0.8384
RSimpson	-0.010	0.164	0.019	0.042	0.092	0.222	0.223	0.010	RSimpson	0.9776	0.6506	0.9586	0.9073	0.8013	0.5381	0.5360	0.9775
RAce	0.245	0.085	-0.120	0.190	0.161	-0.016	-0.218	0.247	RAce	0.4945	0.8149	0.7403	0.5981	0.6570	0.9645	0.5452	0.4915
RChao1	0.220	0.074	-0.107	0.172	0.133	-0.019	-0.239	0.218	RChao1	0.5406	0.8391	0.7687	0.6345	0.7142	0.9588	0.5064	0.5454
RShannoneven	-0.015	-0.095	-0.141	-0.077	-0.115	-0.105	0.010	-0.019	RShannoneven	0.9665	0.7932	0.6973	0.8328	0.7526	0.7725	0.9771	0.9579
RSimpsoneven	-0.095	-0.239	0.012	-0.150	-0.200	-0.266	-0.167	-0.098	RSimpsoneven	0.7949	0.5052	0.9747	0.6789	0.5792	0.4573	0.6454	0.7870
RPd	0.108	0.025	-0.200	0.055	0.013	-0.014	-0.073	0.083	RPd	0.7661	0.9452	0.5802	0.8808	0.9715	0.9693	0.8408	0.8187

CON represents the control group, which received milk without sodium butyrate (SB) supplementation. LSB refers to the low SB supplementation group, MSB refers to the medium SB supplementation group, and HSB refers to the high SB supplementation group. Before the diversity index, R represents the ruminal outcome, while I represents the intestinal outcome. Data were considered significant at $p < 0.05$ and $n = 10$.

(**A**)

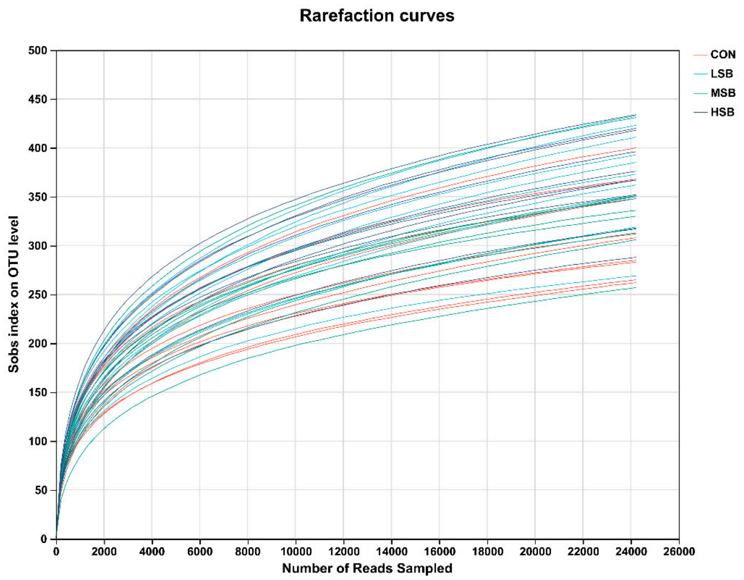

(**B**)

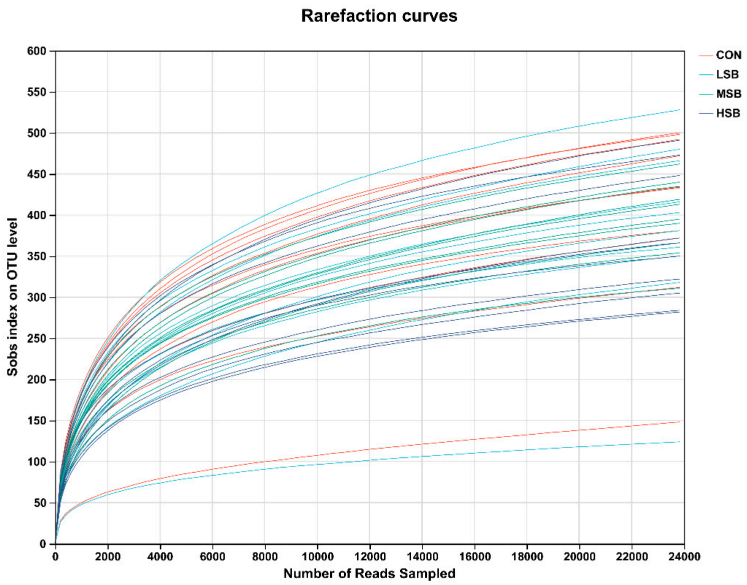

Supplemental Figure S1. Effects of sodium butyrate supplementation on rarefaction curves of ruminal and intestinal microbiota in preweaning calves. (**A**) The ruminal microbiota. (**B**) The intestinal microbiota CON represents the control group, which received milk without sodium butyrate (SB) supplementation. LSB refers to the low SB supplementation group, MSB refers to the medium SB supplementation group, and HSB refers to the high SB supplementation group.
